# Questioning the definition of Tourette syndrome—evidence from machine learning

**DOI:** 10.1093/braincomms/fcab282

**Published:** 2021-12-02

**Authors:** Theresa Paulus, Ronja Schappert, Annet Bluschke, Daniel Alvarez-Fischer, Kim Ezra Robin Naumann, Veit Roessner, Tobias Bäumer, Christian Beste, Alexander Münchau

**Affiliations:** 1 Institute of Systems Motor Science, University of Lübeck, 23562 Lübeck, Germany; 2 Department of Neurology, University of Lübeck, 23538 Lübeck, Germany; 3 Cognitive Neurophysiology, Department of Child and Adolescent Psychiatry, Faculty of Medicine, TU Dresden, 01069 Dresden, Germany; 4 Faculty of Medicine, University Neuropsychology Centre, TU Dresden, 01069 Dresden, Germany; 5 Cognitive Psychology, Faculty of Psychology, Shandong Normal University, Qianfoshan Campus, No. 88 East Wenhua Road, Lixia District, Jinan, 250014, China

**Keywords:** Tourette syndrome, machine learning, video scoring

## Abstract

Tics in Tourette syndrome are often difficult to discern from single spontaneous movements or vocalizations in healthy people. In this study, videos of patients with Tourette syndrome and healthy controls were taken and independently scored according to the Modified Rush Videotape Rating Scale. We included *n* = 101 patients with Tourette syndrome (71 males, 30 females, mean age 17.36 years ± 10.46 standard deviation) and *n* = 109 healthy controls (57 males, 52 females, mean age 17.62 years ± 8.78 standard deviation) in a machine learning-based analysis. The results showed that the severity of motor tics, but not vocal phenomena, is the best predictor to separate and classify patients with Tourette syndrome and healthy controls. This finding questions the validity of current diagnostic criteria for Tourette syndrome requiring the presence of both motor and vocal tics. In addition, the negligible importance of vocalizations has implications for medical practice, because current recommendations for Tourette syndrome probably also apply to the large group with chronic motor tic disorders.

## Teaser

Paulus et al. report which aspects of Tourette syndrome phenomenology are most useful for diagnosing Tourette syndrome. Using a machine learning-based analysis, they show that the severity of motor tics, but not vocal tics, is the best predictor to separate patients with Tourette syndrome and healthy controls.

## Graphical Abstract

**Graphical Abstract:**
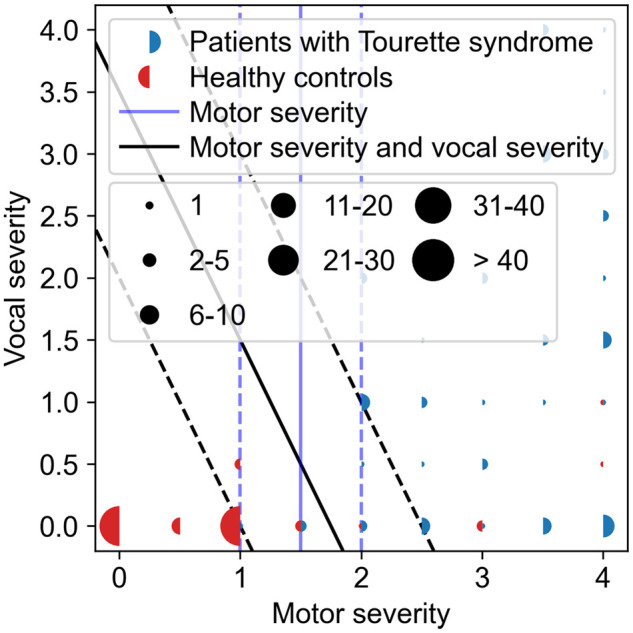

## Introduction

Tourette syndrome is a common neurodevelopmental disorder, diagnosed when an individual has both motor and one or more vocal (phonic) tics with an onset before the age of 18 years and a duration of more than 1 year.^1^ Reported prevalence rates vary widely across studies; the prevalence rate in children is estimated to be 0.3–0.9%.^2^ Lifetime prevalence of tics though is probably higher. In cases, where tics have lasted for less than a year, a diagnosis of provisional tic disorder can be made. In most of these patients, tics persist for more than a year, but tic severity is usually minimal in them.^3^ While definition and categorization of Tourette syndrome appear clear and unambiguous, clinical diagnosis is not always straightforward because tics resemble spontaneous movements or vocalizations often seen in healthy people. Even for Tourette syndrome experts, it is sometimes difficult to reliably discern tics from single spontaneous movements in healthy people.^4^ Although vocal tics are considered a ‘conditio sine qua non’ for diagnosing Tourette syndrome,^1^ motor symptoms often prevail.^5^ As yet, it has not been rigorously examined though, which aspects of Tourette syndrome phenomenology are most useful (predictive) in diagnosing an individual with Tourette syndrome. To address this, we applied machine learning to independent video ratings of motor and vocal tics using the well-established Modified Rush Videotape Rating Scale (MRVRS).^5^ Videos were also taken from healthy controls and assessed by a group of Tourette syndrome experts.

## Materials and methods

As part of a study of neural mechanisms underlying Tourette syndrome,^6^^,^^7^ we recruited *n* = 101 patients with Tourette syndrome and *n* = 111 healthy controls. A diagnosis of Tourette syndrome was made according to diagnostic criteria of Diagnostic and Statistical Manual of Mental Disorders, Fifth Edition (DSM 5), after a thorough clinical assessment as described previously.^6^ A standardized video of all participants was taken using the MRVRS.^5^ According to the protocol,^5^ we scored (from 0 to 4) in five categories: number of body areas, frequency of motor/vocal tics, severity of motor/vocal tics yielding a total score ranging from 0 to 20.^5^ In addition, motor tic count per minute was computed.^6^ Each video (patients and healthy controls) was independently scored by two clinicians experienced in the assessment of Tourette syndrome. On many videos of healthy controls spontaneous particularly facial movements occurred that could not reliably be distinguished from tics. These extra movements were counted as tics. When Rush total scores or tic counts differed by >15%, videos were reviewed and discussed by the two raters to determine a Rush consensus score and tic counts differing by less than 15% in all cases. All participants, as well as the legal guardians of children and adolescents, provided written informed consent for video recording. The study was approved by the Ethics Committee of the University of Dresden (EK 359092017).

### Data analysis

We conducted a support vector machine (SVM)-based analysis to examine the impact of each category of the MRVRS, the motor tic count per minute, as well as age and gender, for the classification of individuals into the groups ‘Tourette syndrome’ and ‘no Tourette syndrome’. To eliminate the effect of different ranges of features, we first normalized all features into a *z*-score. To rank the features regarding their relevance to the classification, we trained an SVM classifier with linear kernel on the whole dataset. SVM uses a hyperplane to separate the instances into the two groups. The normal to this separating hyperplane is the vector of weights *w* = ($w_{1}$,…,$w_{d}$). The values |$w_{i}$| were used for the feature ranking. Each |$w_{i}$| reveals how strong the corresponding feature contributes to the classification.^8^ Starting with the feature with the highest impact and consecutively adding the next feature, we trained SVMs with linear kernel, cost equal to 5 and gamma equal to 10^−7^. The trained SVMs were then used to classify each individual into the two groups. Given the number of individuals we used *k*-fold cross-validation with *k* = 10 on the results of the SVM. The classification accuracy for each feature was calculated as the mean of all 10 repetitions. Using that data, we also calculated the 99% confidence interval for each feature set to examine whether the addition of features led to an increase in classification accuracy in the two diagnostic categories (i.e. ‘Tourette syndrome’ versus ‘no Tourette syndrome’). No overlap between the calculated 99% confidence bounds indicates a significant increase in classification accuracy. Since the feature ranking and the evaluation were done in the same dataset, we conducted further analysis to estimate generalization. Therefore, we divided the data in a training set (70%) and a validation set (30%). We applied the feature ranking and the *k*-fold cross-validation analysis on the training set. Then, we trained a SVM on the whole training set and used it to classify the validation set to calculate validation accuracy. Moreover, we used permutation tests for the features ranked in the training data and employed it on the validation set. Therefore, the data were randomly divided into two groups and SVM was used to predict these random group labels. We repeated this process 1000 times and calculated the number of times where the features predicted ‘Tourette syndrome’ or ‘no Tourette syndrome’ better than randomly assigned labels. To verify the importance of the selected features for the validation accuracy, we trained SVMs without the best features on the training set and used them for prediction on the validation set.

### Data availability

Anonymized data can be shared by request from any qualified investigator.

## Results

Demographical data, MRVRS variables and motor tic count per minute are given in Table 1. The SVM analysis of the video scores showed that 13 healthy controls were outliers (detected via interquartile range method)^9^ in one or more variables of the MRVRS. Videos of these participants were re-assessed by the two raters and another not previously involved Tourette syndrome expert. Based on clinical phenomenology they decided whether any of these outliers would possibly or likely be diagnosed with Tourette syndrome. Two of the 13 healthy controls were independently rated as likely Tourette syndrome and were therefore excluded from further analyses.

**Table 1:** Demographic data and variables of the MRVRS, as well as motor tic count per minute separately for the patient and control group.

Variables	Patients with Tourette syndrome	Healthy controls
Age^a^	17.36 ± 10.46	17.62 ± 8.78
Gender (*n*), males: females	71:30	57:52
Disease duration (years)^a^	9.9 ± 9.4	–
Rush, number of body areas^a^	3.11 ± 1.01	0.84 ± 0.88
Rush, motor tics frequency^a^	2.31 ± 1.13	0.71 ± 0.70
Rush, vocal tics frequency^a^	0.81 ± 0.84	0.02 ± 0.12
Rush, motor tics severity^a^	3.08 ± 0.92	0.76 ± 0.79
Rush, vocal tics severity^a^	1.14 ± 1.22	0.02 ± 0.12
Rush, total score^a^	10.45 ± 4.14	2.34 ± 2.39
Motor tic count per minute^a^	39.20 ± 29.25	7.35 ± 12.12

a Mean values and standard deviations are given.

**Figure 1.**
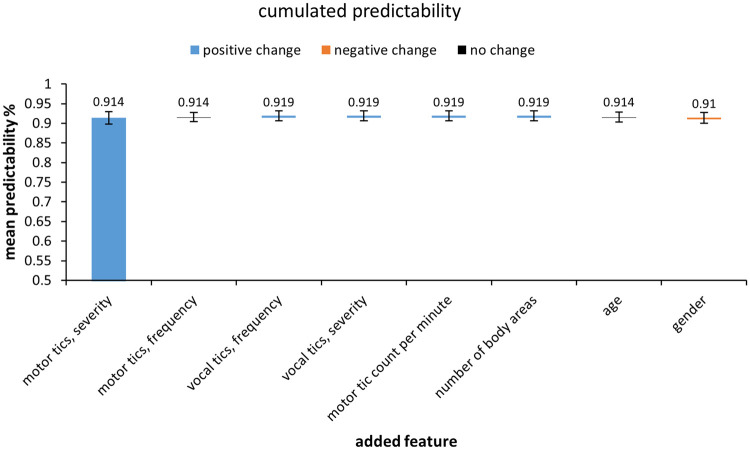
**Results from the *k*-fold cross-validation analysis.** The first bar shows the classification accuracy of the first feature (motor tics severity of the MRVRS). The other bars indicate if and how much the cumulated classification accuracy increased compared to the best feature. Blue indicates a positive change, orange a negative change and black no change in classification accuracy. The 99% confidence intervals are displayed as black error bars.

Figure 1 shows the results of the SVM analysis. The first bar shows the prediction accuracy of the feature contributing most to SVM classification performance. This first feature was motor tics severity. The other bars reveal if and how much the cumulated predictability increased when adding more features. The error bars indicate the 99% confidence intervals. Motor tics severity already led to a prediction accuracy of 91.4% (chance level = 50%). Adding more features to the analysis did not significantly improve predictability as indicated by the overlapping 99% confidence bounds.

To evaluate the generalization ability of the feature ranking we conducted an additional analysis with separate training and validation sets. The results of this analysis are shown in Table 2. The ranking of the features is quite similar to the feature ranking in the first analysis and there is no change in the three most important features. The training accuracy as well as the validation accuracy for the best feature is 92% and neither improves by adding more features. In all of the permutation tests, the validation accuracy of the real groups (‘Tourette syndrome’, ‘no Tourette syndrome’) was higher than the prediction accuracy of the random labels. Consecutively, omitting the best features led to a significant decrease of validation accuracy. The validation accuracy drops to, or below, chance level when all variables of the MRVRS are omitted and just gender and age remain.

**Table 2:** Summary of the results of the additional SMV analysis.

Added feature	**Accuracy in training set**^a^ (%)	**Accuracy in validation set**^b^ (%)	**% in which the prediction is better than randomly assigned labels**^c^ (%)	**Validation accuracy without selected features**^d^ (%)
Rush, motor tics severity	92	92	100	86
Rush, motor tics frequency	91	92	100	87
Rush, vocal tics frequency	91	92	100	87
Rush, number of body areas	91	92	100	83
Rush, vocal tics severity	91	92	100	79
Motor tic count per minute	91	92	100	59
Gender	91	92	100	44
Age	91	92	100	50

a The first column contains the consecutively added or removed features for the additional SVM analysis ranked on the training set.

b The second column gives the cumulated training accuracy validated through 10-fold cross validation.

c Cumulated validation accuracy is displayed in the third column. The third column indicates in how many out of 1000 permutation tests the prediction of the true group labels were more accurate than the prediction of randomly assigned labels.

d The last column shows the accuracy on the validation set reached by a SVM trained without the given features. For example, in row six it can be seen that omitting the best six features in training lead to a validation accuracy of 59%.

## Discussion

The main finding of this study is that although clinical phenomenology of Tourette syndrome is multi-faceted, only a single category of the MRVRS, i.e. the severity of motor tics, is sufficient to classify an individual as having Tourette syndrome with an accuracy of >90%. This is of great relevance for the conceptualization of Tourette syndrome and clinical practice. It challenges the biological validity of current diagnostic criteria for Tourette syndrome requiring the presence of both motor and vocal tics.^1^ It appears that the severity of motor tics, i.e. the amount of extra movements, is far more relevant for the grouping of Tourette syndrome according to DSM 5 than vocal phenomena. Although, clearly, there are patients with Tourette syndrome, who develop severe vocal tics including coprolalia in the course of their disease, vocalizations seem to have a negligible importance for the categorization of Tourette syndrome. This interpretation is also very relevant for the public (mis-)conception of Tourette syndrome as a disorder mainly presenting with vocalizations including coprolalia.^10^

In conclusion, the SVM analysis presented here suggests that vocal tics are not a ‘conditio sine qua non’ for the diagnosis of Tourette syndrome questioning the validity of the current Tourette syndrome definition according to DSM 5. As a consequence, it lends support to recent recommendations and data suggesting that Tourette syndrome and chronic motor tic disorder should not be separated as distinct disorders^11–13^ but rather that chronic motor tic disorder is a less severe and Tourette syndrome a more severe manifestation of a continuous neurodevelopmental tic spectrum disorder.^12^^,^^13^ This, in turn, has implications for medical practice, because current recommendations for Tourette syndrome would then also apply to the group of chronic motor tic disorders. This is supported by the results of recent clinical studies using statistical techniques such as hierarchical cluster analysis. According to these, Tourette syndrome is a condition with multiple phenotypes rather than a unitary condition as implied by current diagnostic criteria.^14^^,^^15^

## Abbreviations

DSM 5 =: Diagnostic and Statistical Manual of Mental Disorders, Fifth Edition
MRVRS =: Modified Rush Videotape Rating Scale
SVM =: support vector machine
